# Ethanol Conditioned Taste Aversion in High Drinking in the Dark Mice

**DOI:** 10.3390/brainsci9010002

**Published:** 2019-01-01

**Authors:** John C. Crabbe, Pamela Metten, Antonia M. Savarese, Angela R. Ozburn, Jason P. Schlumbohm, Stephanie E. Spence, Wyatt R. Hack

**Affiliations:** Portland Alcohol Research Center, Department of Behavioral Neuroscience, Oregon Health & Science University and VA Portland Health Care System, Portland, OR 97239, USA; mettenp@ohsu.edu (P.M.); savarese@ohsu.edu (A.M.S.); ozburn@ohsu.edu (A.R.O.); schlumbo@ohsu.edu (J.P.S.); spences@ohsu.edu (S.E.S.); hack@ohsu.edu (W.R.H.)

**Keywords:** binge drinking, aversion, selective breeding, pharmacogenetics, inbred strains, availability, concentration, thirst motivation, animal model

## Abstract

Two independent lines of High Drinking in the Dark (HDID-1, HDID-2) mice have been bred to reach high blood alcohol levels after a short period of binge-like ethanol drinking. Male mice of both lines were shown to have reduced sensitivity to develop a taste aversion to a novel flavor conditioned by ethanol injections as compared with their unselected HS/NPT founder stock. We have subsequently developed inbred variants of each line. The current experiments established that reduced ethanol-conditioned taste aversion is also seen in the inbred variants, in both males and females. In other experiments, we asked whether HDID mice would ingest sufficient doses of ethanol to lead to a conditioned taste aversion upon retest. Different manipulations were used to elevate consumption of ethanol on initial exposure. Access to increased ethanol concentrations, to multiple tubes of ethanol, and fluid restriction to increase thirst motivation all enhanced initial drinking of ethanol. Each condition led to reduced intake the next day, consistent with a mild conditioned taste aversion. These experiments support the conclusion that one reason contributing to the willingness of HDID mice to drink to the point of intoxication is a genetic insensitivity to the aversive effects of ethanol.

## 1. Introduction

Binge-like drinking at a young age is a substantial risk factor for alcohol use disorder [1]. Binge drinking is also highly prevalent in the USA [2] and confers many adverse health and socioeconomic consequences [3]. One common definition of binge drinking is a time-limited pattern of ingestion that leads to blood alcohol levels (BALs) in excess of 80 mg% [4]. Binge-like patterns of drinking are common among individuals with a diagnosis of alcohol use disorder and/or alcohol dependence, although these three describe distinct entities and may have different implications for which therapeutic approaches may prove to be effective [5]. There are many mouse and rat lines available that have been selectively bred for high preferential ingestion of 10% ethanol (EtOH) vs. tap water when both fluids are continuously present in the home cage. The rat lines typically ingest 5–7 g EtOH/kg body weight/day [6], while the mouse lines drink more, between 14–23 g/kg/day [7]. Except under certain procedural conditions and/or without extensive experience, these animals generally do not pattern their drinking such that they reach BALs leading to behavioral intoxication [8]. For example, even mice genetically predisposed to ingest EtOH solutions (notably, mice of the C57/C58 Group 4 lineage [9] generally pattern their drinking to avoid sustaining intoxicating blood alcohol levels [10]. This somewhat limits their utility to study alcohol’s pathobiology, as many individuals with harmful patterns of drinking engage in binge-like drinking [11].

Drugs of abuse have both rewarding and aversive effects depending on dose, chronicity of use, and timing [12,13]. Unlike many abused drugs, alcohol is ingested orally, and one therapeutic approach is to condition an aversion to the taste of the drug by pairing drinking with nausea and vomiting induced by a different, emetic drug, such as lithium chloride or ipecac (conditioned aversion therapy) [14,15]. This therapy uses the principles of taste aversion conditioning, which has been demonstrated in many species. The Pavlovian conditioning designs employed with rodents postulate that some internal (interoceptive) experience of an injected drug is unpleasant for the animals. If such injections are systematically paired with their experience of the taste of a novel flavored solution, a conditioned aversion to that novel flavor will gradually develop. In the case of ethyl alcohol injections given shortly after ingestion of a weak saline or saccharin solution, this is a conditioned taste aversion (CTA) that is conditioned by alcohol, but not to the taste of alcohol *per se*. Assays based on taste conditioning are commonly used to study the aversive effects drugs of abuse in rodents [13,16]. Some rodents are genetically predisposed to develop a CTA, and a rat line has been bred to be prone to develop a taste aversion to saccharin conditioned by lithium chloride injections [17]. When naive animals from subsequent generations were tested for their preference to drink alcohol solutions vs. water, Taste aversion prone rats drank less alcohol than taste aversion resistant rats [18,19]. Taste aversion prone rats were also found to be sensitive to an ethanol-conditioned taste aversion to a saccharin solution, while the resistant rats failed to develop an ethanol-CTA [20]. This latter finding suggests that some genes promoting good taste aversion learning also predispose animals to avoid drinking ethanol solutions. A review of the genetic contributions to ethanol-CTA learning suggests that the paradigm reflects ethanol’s aversive effects and that studies of ethanol-CTA may reveal how such learned aversions may lead certain genotypes to avoid drinking ethanol [21].

Why humans will continue to drink after they are already intoxicated, but most rodents, even those genetically susceptible to generally high alcohol intake, seem to be unwilling to continue to drink past this threshold is unknown. Whether a rodent achieves a high BAL is a joint function of availability, the amount it ingests, and the pattern of its ingestion; drinking patterns are usually described as consisting of “bouts,” each representing a period when behavior is focused on, and restricted to, drinking. It is also unknown how drinking parameters such as bout size, frequency, and inter-bout interval combinatorially determine a high BAL, though some studies of ingestive behavior [22,23] and particularly of binge-like drinking in mice [24] have been informative. 

To study binge-like drinking in mice, we developed a simple laboratory procedure under which C57BL/6J mice would drink sufficient EtOH during a 2–4 h period early during their circadian dark cycle to achieve BALs exceeding 80 mg%, the level established by the National Institute on Alcohol Abuse and Alcoholism to define a binge [4]. This procedure (drinking in the dark) [25] has since been widely used by the alcohol research community as a tractable model to study the underlying neurobiology and test novel pharmacotherapies [26,27,28].

Using a two-day variant of the drinking in the dark assay, we have selectively bred two independent lines of High Drinking in the Dark (HDID-1, HDID-2) mice to reach high BALs after a short period of binge-like EtOH drinking. When we developed the HDID mouse lines, we elected to remain agnostic as to behavioral mechanisms (e.g., amount ingested, patterning) and to select for breeders those individuals with the highest BALs at the end of a drinking in the dark session [29,30]. Interestingly, the two lines, selected under virtually identical laboratory and husbandry conditions, both achieved high BALs after drinking in the dark, but differ in their drinking microstructures [31] While the amount of EtOH ingested is systematically recorded, it is not taken into account when choosing animals to produce the next generation. Nonetheless, both HDID lines showed substantial increases in the ingested EtOH dose as a correlated response to selection [29,30]. The lines drink to the point of behavioral intoxication [29] and show mild withdrawal convulsions [30] after a binge. After 38 (HDID-1) and 33 (HDID-2) selected generations, these mice now reach BALs in excess of 200 mg% in the limited-access, drinking in the dark test [32]. Because we saw no signs of reaching selection limits, to stabilize the lines, starting with selected generation S26, we began to establish an inbred HDID-1 line through brother-sister matings. The inbred (iHDID-1) line is currently in inbred generation F18. An iHDID-2 line was started in S26 and is in F8. iHDID-1 now average BALs of about 100 mg%, which is consistent with the generation from which it was initiated. Both inbreeding and continued selective pressure are maintained on iHDID-2, so they have stayed at 170 mg% or greater BALs (unpublished data).

Searches for correlates of selective breeding have compared HDID-1 and/or HDID-2 mice with the founder HS/NPT (HS) population [for review, see [33]]. On most traits, no systematic differences from HS have been seen in either selected line; importantly, no differences in EtOH clearance have emerged. However, there are some differences that may be of importance. For example, HDID-1 mice tend to drink in larger bouts, while HDID-2 mice ingest alcohol more frequently during a DID test than HS [31]. EtOH, like other drugs of abuse, is well known to produce both rewarding and aversive subjective effects, depending on the conditions of the conditioning test employed and the species and genotype of the subjects [34,35]. Notably, male mice of both lines were found to have reduced sensitivity to develop a CTA to a novel flavor paired with EtOH injections as compared with HS [36]. There are multiple potential interpretations of conditioned aversions to drugs of abuse [13]. The lines did not differ from HS in their EtOH-induced conditioned place preference, a different Pavlovian conditioning task assumed to reflect a drug’s rewarding effects [21,34]. Thus, our current hypothesis is that while the HDID lines do not show enhanced sensitivity to EtOH’s rewarding subjective effects, they have reduced specific sensitivity to the aversive effects of EtOH [36]

The first goals of the current experiments were to establish whether reduced EtOH-CTA was retained during the process of creating the inbred variants. We also wanted to know whether the original findings in males also characterized females. One limitation of the standard paradigm we use to elicit and test an EtOH-CTA is that it relies on post-ingestion injection of EtOH [37]. While this allows us to control the experienced dose of EtOH, when animals drink in the drinking in the dark test their ingested dose varies more widely, depending on their individual patterns of drinking across time [31,38]. Proclivity to ingest EtOH solutions depends on physiological state (e.g., water balance) and sensory abilities (e.g., taste). Thus, our second goal was to see whether HDID mice could ingest sufficient doses of EtOH to lead to a CTA of the ingested solution itself upon retest. Different manipulations were used to elevate consumption of EtOH on initial exposure. Access to higher EtOH concentrations is one method to induce mice to drink to higher doses (assessed by BAL) [39,40]. Another method is to offer multiple bottles of EtOH at the same concentration. For example, C57BL/6J mice were shown to ingest more EtOH when three tubes of EtOH were offered along with three tubes of water than they drank if a single tube of each fluid was offered [41]. And, of course, fluid restriction to increase thirst motivation enhances initial drinking of EtOH in nearly all tests [39,42]. An early procedure upon which we based initial studies of drinking in the dark employs addition of saccharin, a sweet tasting substance, to EtOH to increase intake [43]. To address our second question, we employed several such manipulations, excluding saccharin adulteration.

## 2. Materials and Methods

### 2.1. Mice and Husbandry

All animals were bred and maintained in the VA Portland Health Care System Veterinary Medical Unit in standard shoebox polycarbonate cages with stainless steel wire bar tops with a recess for chow on Bed-o’cobs^®^ bedding (Andersons, Maumee, OH, USA) changed once weekly. We maintain breeders and offspring at 21 ± 1 °C on a reversed, 12 h:12 h light:dark cycle with lights off at 09:30 AM. *Ad libitum* Purina 5LOD chow (PMI Nutrition International, Brentwood, MO, USA) was available at all times except for two, 20-min periods in Experiment 4. From weaning until the beginning of experiments mice were group housed 2–5/cage. Before the start of each study, mice were habituated to single housing for at least one week. For Experiments 1 and 4, mice were also habituated to a forward light:dark cycle with lights on at 06:00 AM. Sexes, numbers, genotypes and range of selected generations and/or subsequent generations of inbreeding are specified for subjects of each experiment. All mice were between the ages of 52–151 days old at the start of testing. All procedures were approved by the local Institutional Animal Care and Use Committee (Approval #3879-17) and were conducted in accordance with the NIH Guidelines for the Care and Use of Laboratory Animals [44].

### 2.2. Experiments and Methods

#### 2.2.1. Experiment 1. EtOH Conditioned Taste Aversion in Inbred HDID Mice

To see whether the iHDID lines are less sensitive than HS mice to EtOH-induced CTA, male and female iHDID-1, iHDID-2 and HS/NPT mice (116 ± 2 days old: range = 71–151 days) were tested for development of EtOH-CTA using our previously published [36] slight variant of the protocol developed by Dr. Chris Cunningham [37]. iHDID-1 mice were from generation S26F17, iHDID-2 were from S26F7-8, and HS were from generation G92. After one week of adaptation to single housing and a forward light cycle, randomly-assigned groups of 8–12 mice per genotype, sex, and dose were weighed and then restricted to a 2 h period of unmeasured access to water for 1 week during hours 2.5–4.5 after lights on. This habituated the mice to a scheduled period of fluid access. Mice were weighed daily and any animal with >15% weight loss from its weight prior to water scheduling was supplemented with an additional 1 h water access 8 h after lights on. To start the tests (initial exposure day), a single tube of 0.2M NaCl was offered for 1 h and intake was measured. This served to acquaint the animals with the novel flavor. The next day, measured water was offered for two hours at the same time of day. The last 10 days comprised the conditioning phase of the study. Days 1, 3, 5, 7, and 9 were conditioning days where 1 h measured access to 0.2 M NaCl was offered. Each of the 5 NaCl conditioning trials was immediately followed by ip administration of either vehicle (0.9% NaCl) or EtOH (2.0, 3.0, or 4.0 g/kg: 200 proof, Decon Labs, King of Prussia, PA, USA, 20% *v*/*v* in vehicle). Four hours later, all animals were provided with unmeasured water for 30 min. On Days 2, 4, 6, 8, and 10, mice were given 2 h measured access to water. The final conditioning test (Day 11) was conducted offering measured access to 0.2M NaCl fluid for 1 h. Two hundred twenty mice entered the study, and 13% were removed during the procedure for weight loss >20% of baseline. Final numbers of mice/group are given in figure legends. 

#### 2.2.2. Experiment 2. Concentration-Dependent Initial Intake of EtOH: Low Concentrations

For this experiment, we asked whether the first EtOH drinking experience (with either 5, 10, 15, or 20% EtOH offered) altered intake of 20% EtOH on subsequent days. We predicted that intake of the higher concentrations would lead to higher ingested doses on Day 1 and then to lower intake on subsequent days. Four groups of 13–14 female HDID-1 mice (54–87 days old) from selected generation S25 were offered a single bottle of EtOH in a four-day drinking in the dark test. Days 1–3 were measured with 2 h access. Day 4 was measured with 4 h access, immediately after which a blood sample was taken to assess BALs. On Day 1, mice were offered either 5, 10, 15, or 20% EtOH (*v*/*v*); all mice were offered 20% EtOH on Days 2–4.

#### 2.2.3. Experiment 3. Concentration-Dependent Initial Intake of EtOH: High Concentrations 

We reasoned from the results of Experiment 2 that the BALs achieved on Day 1 might have been insufficient to elicit a CTA. Furthermore, in Experiment 2, three of the four groups of mice were asked to express any conditioned effect by responding to a higher concentration of EtOH on subsequent days. In Experiment 3 we offered 20%, 30%, or 40% concentrations to separate groups of mice for both test days of a two-day drinking in the dark experiment. Six groups (*n* = 10–12 per sex/line/EtOH concentration) of male and female HDID-1 (generation S37–38) and HDID-2 (generation S32) mice, 77–139 days old, were tested with 2 h exposure on Day 1 and 4 h on Day 2, followed by a blood sample for BAL. 

#### 2.2.4. Experiment 4. Concentration-Dependence of CTA with Thirst Motivation 

This procedure was modeled on that previously used with C57BL/6J and DBA/2J mice [39]. Groups of HDID-1 (S29–32), HDID-2 (S23–27), and HS/NPT males and females, 54–107 days old, were habituated to individual housing for seven days. Mice were then restricted to 90 min access to water for four days at 3 h after lights off. The next day, food was removed and they were offered 0, 5, 10, 15, or 20% EtOH (*v*/*v*) for 20 min. Tubes were then removed and food was restored. After three more days of 90 min restricted access to water, they were again offered access to their respective concentration of EtOH or water for 20 min in the absence of food. Body weights were monitored daily in this experiment; animals that lost >20% of their Day 1 body weight were removed from the study and euthanized. This occurred in 18/297 mice (6%). Data for the 55 water drinking animals are not shown, leaving *n* = 223 ethanol drinking mice. 

#### 2.2.5. Experiment 5: Effect of Number of Tubes of EtOH on Day 1 

Only male HDID-1 from generation S24 were available for this study. Groups of 14–15 mice per tube number treatment group, 52–102 days old were offered 1, 2, or 3 tubes of 20% EtOH (*v*/*v*) during 2 h sessions on two successive days and ingestion volumes were recorded. (*nb*: This experiment was originally designed for another purpose, and drinking in the dark tests were also given for two more days, plus four more days the following week. As the other results were not informative for the question at hand here, we do not report these data, but they are available upon request).

### 2.3. Statistical Analyses

We generally employed multifactorial ANOVAs and followed up significant interactions with smaller factorial (or one-way) ANOVAs and/or appropriate Tukey’s HSD *post hoc* tests (SYSTAT V 13.1). For the drinking in the dark tests, we first examine all data to look for outliers with spuriously high or low intake. Our experience shows that tubes sometimes leak, and that sometimes an animal will apparently “play” with the tube spout without ingesting the solution. For example, an apparent intake ≥3 g/kg EtOH in 20 min would yield a BAL sufficient to anesthetize the animal if actually drunk. Occasionally, a spurious tube reading leads to a negative “consumption” score, an obvious impossibility. We examine extreme-scoring animals and after comparing their g/kg intake with those in the same genotype, sex, and treatment condition by the studentized residual from regression of BAL on g/kg, for example, an occasional outlier is removed from the data as noted below [45]. We have described in detail elsewhere our rationale for and experience with identifying outliers in limited access drinking (see Supplementary Online Material from [29]). Other statistics are described in the experiments where they were employed. All data are available upon request.

## 3. Results

### 3.1. Experiment 1. Insensitivity to EtOH CTA in iHDID-1 and iHDID-2 Mice

We generally employed the same analysis strategy we have reported earlier [36]. While sexes differed significantly in some analyses, these differences reflected only the well-known greater drinking of females than males. Because sex interacted significantly with any other factor only rarely and non-systematically, we report results of analyses collapsed on sex. Data for the initial NaCl habituation exposure day are not shown and were not analyzed: we first analyzed NaCl intake on the first conditioning day (Day 1), as drinking on this day had not yet been associated with a subsequent injection. We found that Genotypes differed significantly (F2,179 = 35.4, *P* < 0.0001) because iHDID-2 mice drank more saline fluid than either iHDID-1 or HS mice (*Ps* < 0.0001); iHDID-1 drank slightly more than HS (*P* < 0.05). Neither future dose nor its interaction with genotype was significant (Fs < 1), ensuring that each Genotype X Dose group started the conditioning phase with comparable opportunity to develop a CTA. 

**Figure 1:**
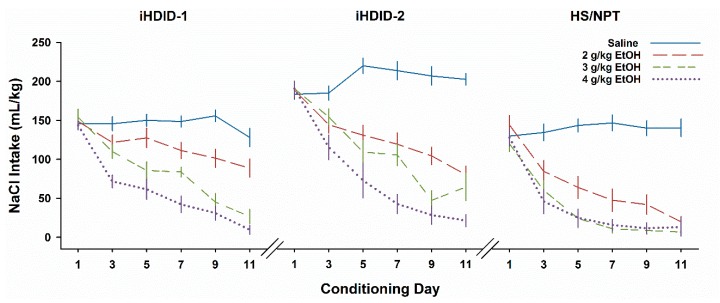
Intake (mL/kg) across days of EtOH taste aversion conditioning varies as a function of genotype and dose. Water intake on even-numbered days is not shown. Mean ± SEM for groups of 12–22 mice per genotype per dose (see Supplemental Figure S1 for sex-specific data).

To assess the development of a CTA, we analyzed Days 3, 5, 7, 9, and 11 with a repeated-measures ANOVA. There was a significant 4-way interaction (F24,668 = 1.6, *P* < 0.05). Results are shown collapsed on Sex in Figure 1 (the patterns for the sexes separately were quite similar and are shown in Supplemental Figure S1). Intake declined significantly across Days (F4,716 = 87.1, *P* < 0.0001). There was also a significant effect of dose (F3,179 = 102.6, *P* < 0.0001), and a significant interaction of Genotype X Dose X Day (F24,716 = 2.3, *P* < 0.001). Inspection of the data suggested that our findings resembled those we had earlier seen in the segregating genotypes. Mice treated with saline showed little to no change in intake across days, while mice treated with 4 g/kg EtOH quickly reduced their intake over days, consistent with a strong CTA. The most apparent difference among the genotypes was seen at the 2 g/kg dose. We pursued the three-way interaction with separate ANOVAs for each day. For Day 3 (the initial test after a paired injection), there were significant effects of Genotype (F2,179 = 33.6, *P* < 0.0001) and dose (F2,179 = 22.3, *P* < 0.0001), but no significant interaction. Beginning with Day 5, and for all subsequent Days, we compared the 2 and 4 g/kg dose groups. For HS, these two groups did not differ significantly on any day. For iHDID-1 (*Ps* ≤ 0.01) and iHDID-2 (*Ps* < 0.02), the 2 g/kg group drank more than the 4 g/kg group on all days. Whether the iHDID-1 mice developed a significant CTA after 2 g/kg EtOH at all is debatable—the only day on which this group differed significantly from the saline group was Day 5 (*P* < 0.01).

### 3.2. Experiment 2. No Evidence of a CTA after Exposure to Lower EtOH Concentrations

**Figure 2:**
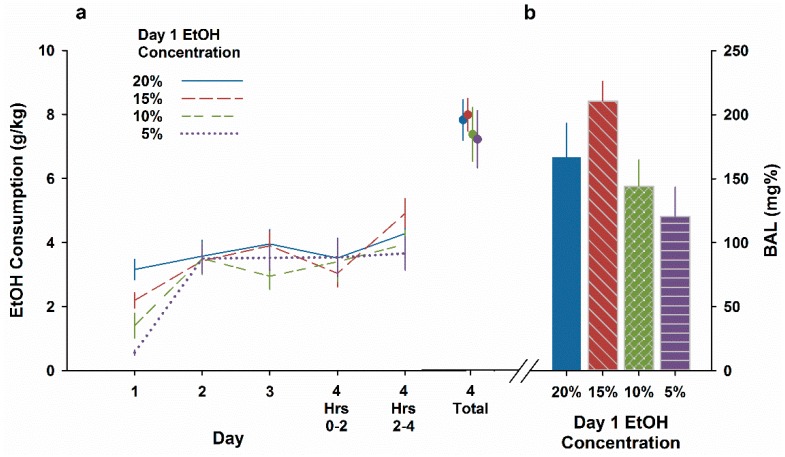
No effect of initial day EtOH dose ingested on subsequent drinking in the dark drinking in HDID-1female mice. (**a**) Drinking for two hour periods on Days 1–4 and total dose ingested in 4 h on Day 4. (**b**) Blood alcohol levels at 4 h on Day 4. Mean ± SEM for groups of 14 mice per Day 1 concentration. All mice drank 20% EtOH on Days 2–4.

We first analyzed drinking during the initial exposure to different EtOH concentrations. There was a clear, ordered concentration-dependent effect on g/kg EtOH ingested intake during the 2 h exposure (F3,51 = 15.3, *P* < 0.001, see Figure 2a). This occurred because all groups consumed nearly equivalent volumes of fluid in mL (Mean ± SEM: 20%, 0.27 ± 0.03; 15%, 0.36 ± 0.11; 10%, 0.34 ± 0.03; 5%, 0.37 ± 0.04). When intakes were expressed as mL/kg body weight (i.e., the effective ingested dose), the groups did not differ (*F* < 1). We next analyzed intake during the 2 h sessions of access to 20% EtOH on days 2–4 with repeated measures ANOVA. Neither day nor concentration, nor their interaction, were significant (all Fs < 1), and intakes ingested closely resembled the intake during 2 h on Day 1 for the 20% group, about 3–4 g/kg. Finally we analyzed total intake ingested during all 4 h of access on Day 4 and found greater total intake in all groups (averaging about 7–8 g/kg), but no significant differences based on Day 1 history (*F* < 1). Blood alcohol levels taken at the end of Day 4 drinking differed significantly among groups (F3,51 = 3.1, *P* < 0.05, see Figure 2b). Mice initially offered 15% EtOH reached higher BALs than those initially offered 5% EtOH (*P* < 0.05.). Overall, this experiment suggested that mice had not achieved a CTA to EtOH they had orally ingested at any dose.

### 3.3. Experiment 3. EtOH CTA after Ingestion of Higher EtOH Concentrations 

**Figure 3:**
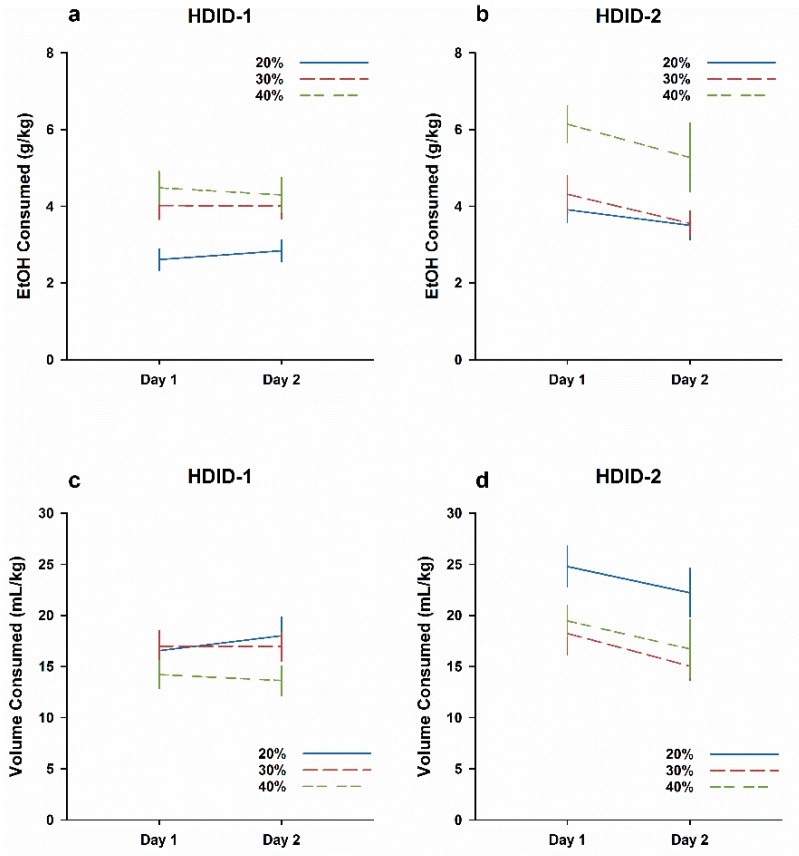
HDID-2 but not HDID-1 mice show a CTA after ingesting higher EtOH concentrations. Mean ± SEM EtOH drinking during the initial 2 h each day is shown for groups of 23–24 mice per genotype and concentration, collapsed on sex. (**a**,**b**) Dose EtOH ingested in g/kg. (**c**,**d**) Volume of 20% EtOH solution consumed in mL/kg.

Results are shown in Figure 3 collapsed across sex. An initial ANOVA of EtOH intake (Genotype × Concentration × Sex × Day) revealed a significant 4-way interaction (F2,129 = 3.6, *P* < 0.05), so genotypes were subsequently analyzed separately. In line with the results of Experiment 2, a three-way ANOVA of g/kg intake in HDID-1 mice revealed a significant effect of Concentration (F2,66 = 8.9, *P* < 0.001), but no main effects of Sex or Day and no significant interaction (Fs < 1: Figure 3a). This concentration-dependent effect of g/kg intake again occurred because all groups consumed similar volumes of fluid in mL/kg, regardless of concentration (Figure 3c; all Fs ≤ 2.6, *Ps* > 0.05). In contrast, HDID-2 mice were sensitive to the differences in EtOH concentration and titrated their consumption accordingly. The ANOVA of g/kg intake in HDID-2 mice revealed significant main effects of concentration (F2,63 = 8.1), sex (F1,63 = 28.3) and day (F1,63 = 14.6, all *Ps* ≤ 0.001), but no significant interaction (Figure 3b). Similarly, analysis of mL/kg intake in HDID-2 mice revealed significant main effects of concentration (F2,63 = 7.4), Sex (F1,63 = 26.8), and Day (F1,63 = 11.6; all *Ps* ≤ 0.001), with no significant interaction (Figure 3d). In contrast to HDID-1 mice, HDID-2 mice showed reductions of EtOH intake on Day 2 relative to Day 1, suggesting an EtOH CTA. Overall, these data suggest that EtOH intake of HDID-2 mice varies as a function of the offered concentration. Further, HDID-2 are susceptible to developing CTA to EtOH when orally ingested, while HDID-1 mice consume the same volume of EtOH regardless of concentration and are resistant to orally-ingested EtOH CTA.

Total ingested dose over the 4 h DID session on Day 2 is shown in Supplemental Figure S2a, and the BAL immediately thereafter in Supplemental Figure S2b. For ingested dose, there was a significant effect only of concentration (F2,136 = 10.4, *P* < 0.0001; all other Fs < 1). HDID-1 reached significantly higher BALs than HDID-2 (F1,137 = 4.2, *P* < 0.05), but neither concentration nor the interaction were significant (both Fs ≤ 2.0, *Ps* > 0.05).

### 3.4. Experiment 4. CTA with Thirst Motivation in HDID-1 and HDID-2 but not HS Mice

**Figure 4:**
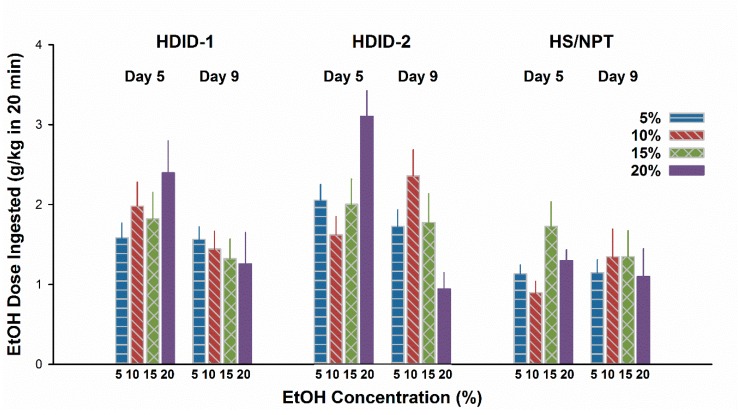
Concentration- and genotype-dependence of initial EtOH ingestion in HDID-1, HDID-2, and HS/NPT mice under conditions of fluid restriction. Mean ± SEM for groups of 16–20 mice per genotype and EtOH concentration.

**Figure 5:**
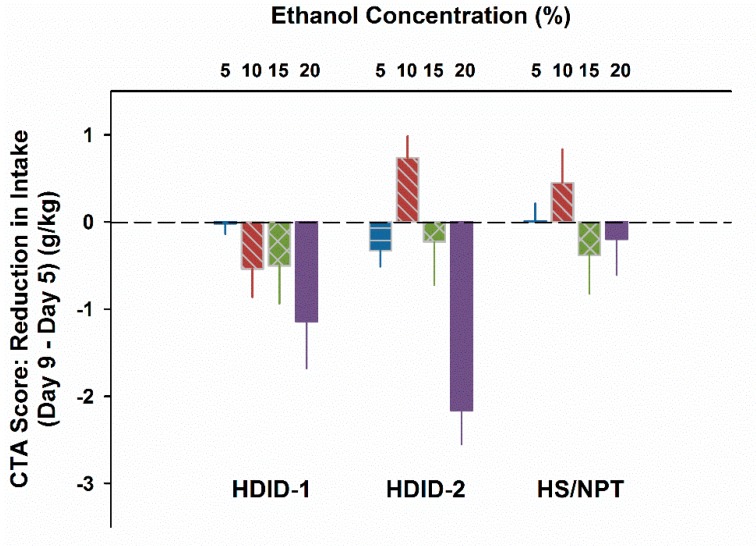
HDID-1 and HDID-2, but not HS/NPT, mice develop an EtOH CTA under conditions of fluid restriction. CTA score = Day 9−Day 5 g/kg EtOH ingested during 20 min. Mean ± SEM for groups of 16–20 mice per genotype and concentration.

For results see Figure 4. On Day 5, mL/kg intake differed as a function of Genotype (F2,263 = 48.7, *P* < 0.0001) and Concentration (F4,263 = 16.8, *P* < 0.0001), but not their interaction. Exclusion of the water drinking animals to analyze g/kg EtOH intake showed the same pattern, except that the interaction term approached significance (*P* = 0.06). Given the different amounts ingested at different concentrations on Day 5, to test for a potential CTA we calculated a difference score by subtracting Day 5 intake from Day 9 intake. This yielded a greater negative value for a more pronounced CTA (Figure 5). These CTA scores differed significantly by concentration (F3,211 = 7.5, *P* < 0.0001); however, the main effect of Genotype was not significant (F2,211 = 2.4, *P* > 0.09). The interaction of genotype by concentration was also significant (F6,211 = 2.8, *P* < 0.02). Based on the pattern of changes shown in Figure 5, we asked whether the CTA scores of the HDID-1 and HDID-2 mice drinking 20% EtOH differed significantly from zero. Post hoc one-sample t-tests showed that both HDID-1 and HDID-2 drank significantly less on Day 9 than on Day 5 (CTA scores < 0; *Ps* < 0.05).

### 3.5. Experiment 5. Multiple Bottle Drinking Leads to a CTA 

**Figure 6:**
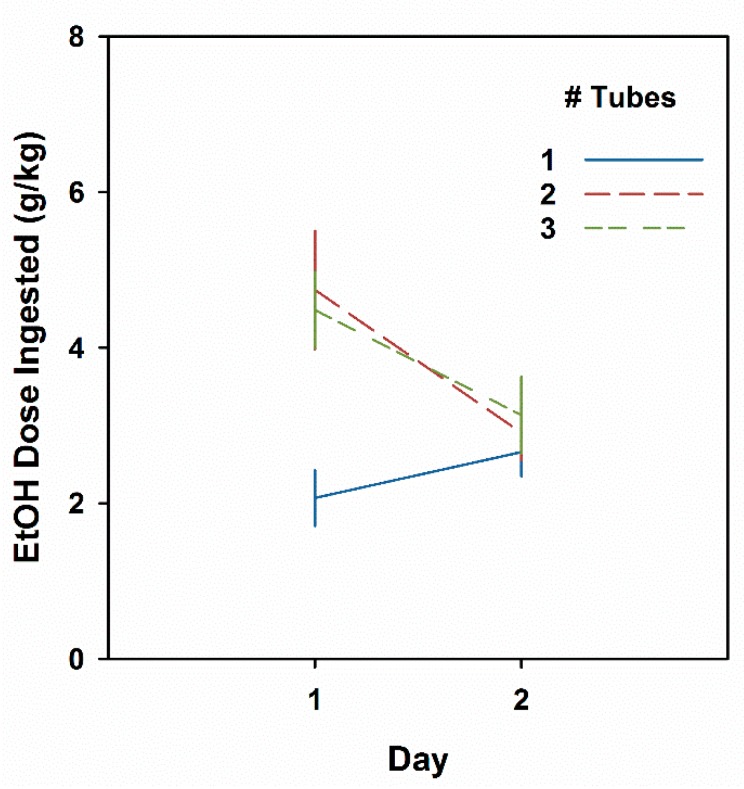
Male HDID-1 mice offered two or three tubes of EtOH drink more than those offered a single tube and show a CTA the next day. Mean ± SEM for groups of 15–16 mice offered one, two, or three tubes of 20% EtOH.

Results are shown in Figure 6. Data from one animal from each of the two- and three-tube groups were eliminated with studentized residual scores of 4.6 and 3.7, respectively. Consumption differed as a function of number of tubes (F2,40 = 4.5, *P* < 0.05) and Day (F1,40 = 8.2, *P* < 0.01), and their interaction (F2,40 = 6.2, *P* < 0.01). On Day 1, mice offered two or three tubes drank more than twice as much EtOH as those offered a single bottle (*Ps* ≤ 0.01) but did not differ from each other. Day 2 intakes were not different among groups (Fs < 1). Analysis of the Day 2 minus Day 1 difference scores showed that both the two- and three-tube groups had significantly reduced intakes on Day 2 (*Ps* < 0.01) when compared with the one-tube group.

## 4. Discussion

We believe that the correlated response to selection in the HDID lines that is most likely to be informative for interpreting their motivation for drinking to excess is their insensitivity, relative to the non-selected HS mice from which they were selected, to develop an EtOH CTA [36]. We hypothesize that they do not experience the aversive effects of intoxication (which they surely achieve). These animals have been fairly widely used to study neurobiological mechanisms of alcohol’s effects, and we wanted to be able to export exemplars from our colonies to other laboratories for more extensive work. However, when we relaxed selection pressure between Generations S28 and S29 in HDID-1 and bred mice without testing for DID, we saw a distinct decline in the BALs of offspring (from about 140 to 120 mg%), so we re-initiated phenotypic testing each generation (selection was never relaxed for HDID-2) [32]. This is consistent with the low heritability (and evident polygenicity) of drinking in the dark BALs in both replicates of the selection [30] We have evidently still not reached the limits of selection [46,47]. We reasoned that other laboratories would be unwilling to undertake the effort required to maintain the selection phenotype, i.e., accept large populations of mice and conduct phenotypic tests of drinking in the dark and BALs each generation to maintain selection intensity in their population.

The solution to this problem was to begin to inbreed each selected line. We, therefore, initiated subpopulations of inbred HDID-1 (iHDID-1) starting with the 26th selected generation for each line. The inbred HDID-1 line is currently in F18, and iHDID-2 is in F8. We did not select while inbreeding HDID-1, so as inbreeding progressed, the phenotype regressed somewhat, and iHDID-1 now average BALs of about 100 mg% (which agrees pretty well with the generation when we started). We have been both inbreeding iHDID-2 and selecting as we go, so they have stayed at 170 mg% or greater without noticeable regression. However, as inbreeding progresses, the loss of allelic diversity typically leads to a reduction in genetic fitness and it can also be accompanied by the changes in previously correlated responses to selection [48] The first major point of the current studies was to test this hypothesis. We found in Experiment 1 that the HS line developed nearly equivalent CTA to all EtOH doses (see Figure 1). The iHDID-1 line clearly has retained insensitivity to an EtOH CTA relative to HS. The iHDID-2 line is also somewhat less sensitive than HS, but the difference is more subtle. This actually agrees rather well with the original finding in the segregating selected lines [compare Figure 1 here with Figures 1 and 2 in [36]]. 

The other principal finding from Experiment 1 is that both males and females of the inbred lines show the blunted CTA response. While the initial findings were in males only, the retention of the CTA difference in the inbred HDID lines strongly suggests that it is also present in the segregating lines as well. There are many potential correlated responses to selection that remain fixed in the inbred lines. We are performing tests of some of them (e.g., determining rate of EtOH elimination, sensitivity to EtOH intoxication), but these studies are not complete. The genetic correlates of drinking in the dark BALs in HDID mice have been reviewed [27,33]. It therefore remains a reasonable speculation that responses known to distinguish both HDID lines from HS have been retained in the iHDID lines, but any such correlate will need to be verified.

While Experiment 1 showed blunted sensitivity in the inbreds, they (and the segregating HDID lines) retain sensitivity to an EtOH CTA if the dose is increased. In Experiment 1 here, and in the earlier test of HDID-1 and -2, both lines showed a nearly complete taste avoidance of saline after 4 g/kg injections [36]. However, the CTA was in all cases to an experimenter-administered subsequent dose of intraperitoneal EtOH. Experiments 2–5 asked in different ways whether we could enhance the oral EtOH intake of the HDID lines during their initial drinking experience so that they displayed reductions in intake during a subsequent EtOH-drinking experience. Experiment 2 showed that when HDID-1 mice were first offered low to modest concentrations of EtOH (5–20% *v*/*v*) for 2 h, they responded by drinking the same amount, regardless of concentration. This engendered an orderly concentration-response relationship for self-administered EtOH on a g/kg basis. However, these self-administered doses of EtOH were apparently unable to produce an aversive interoceptive effect, as the mice in each group drank nearly the same amounts of EtOH on subsequent days. We have no explanation for why the group offered 15% on Day 1 seemed to drink slightly more 20% EtOH than the other groups did in 4 h on Day 4 and achieve an elevated BAL (see Figure 2), but we attribute this to stochastic variation.

This inability of self-administered ethanol to produce an aversive response persisted in the HDID-1 mice, even when concentrations of EtOH were substantially increased in Experiment 3 (Figure 3a,c). Despite achieving BALs well above the level of intoxication (180–230 mg% across groups, Supplemental Figure S2), HDID-1 mice did not differ in EtOH intake when the initial 2 h of Day 2 was compared with Day 1 (Figure 3). In contrast, HDID-2 mice titrated EtOH intake based on concentration, drinking significantly less of the 30% and 40% EtOH (mL or mL/kg) relative to the 20% EtOH solution. HDID-2 mice also exhibited a sensitivity to the aversive properties of EtOH, drinking significantly less early in the session on Day 2 relative to Day 1 (Figure 3b,d). HDID-2 mice consumed a greater volume of EtOH on Day 1 than HDID-1 mice (Figure 3), but the genotypes did not differ in their total ingested EtOH dose on Day 2 (Supplemental Figure S2). Interestingly, despite the genotypes’ ingestion of similar amounts and doses of EtOH on Day 2 (both g/kg and mL/kg), HDID-1 mice achieved significantly higher blood alcohol levels, suggesting that differences in the pattern of intake are physiologically relevant for intoxication. In earlier comparisons of the selected lines with HS mice, we reported that HDID-2 drinking in the drinking in the dark test was characterized by more frequent small bouts than HS, while HDID-1 mice drank in significantly larger bouts [31]. Unfortunately, we cannot ascertain the pattern of drinking in Experiment 3 and so cannot explain with certainty the role of drinking microstructure in its outcome BALs. 

One obvious way to increase initial ingestion of an EtOH (or any other, for that matter) solution is to restrict fluid access. Scheduled access and fluid restriction have been employed, for example, to yield EtOH drinking in mice that can lead to high BALs after drinking for 10 min [42] or 30 min [39,49]. An early exploration of EtOH drinking with restricted fluid access sought to clarify the basis for inbred strain differences in EtOH preference drinking between C57BL/6J and DBA/2J strains. C57BL/6J are well-known to prefer weak alcohol solutions to water, while DBA/2J were known to shun them even at very low concentrations [50]. Belknap used restricted fluid access to motivate drinking during 10-min daily periods in mice of unspecified sex of both genotypes [39]. On the initial exposure day, mice were offered 0, 2, 3, 6, 7, or 10% EtOH and ingested g/kg amounts ranging from 0–3.2 g/kg in generally concentration-dependent fashion. When they were retested four days later, C57BL/6J mice showed significantly attenuated intake of only the 10% EtOH concentration, while DBA/2J mice drank less of 6, 8, and 10% concentrations. Based on the test-retest reductions in intake, as well as the apparently higher sensory detection thresholds reflected in the volume of solution ingested on Day 1, Belknap concluded that C57BL/6J drinking was governed largely by post-ingestive (interoceptive) characteristics, while that of DBA/2J reflected primarily preabsorptive factors, such as taste [39]. 

We modeled Experiment 4 on this report after adapting the fluid restriction and concentration parameters to suit HDID and HS mice. For example, HDID-1 mice readily drink higher concentrations of alcohol than even C57BL/6J [51,52]. At the lower concentrations offered on the initial exposure day (Test Day 1), mice of all three genotypes drank too little to exceed an intake of 2 g/kg (see Figure 4). At the highest (20%) concentration, both HDID-1 and HDID-2 mice ingested >2/g/kg of EtOH and showed significantly lower intake when retested four days later, consistent with development of a CTA. HDID-1 also showed a tendency toward a CTA at the 15 and 10% concentrations. HS mice, as expected, drank significantly less EtOH solution on Test Day 1, and we attribute their failure to show a CTA on Test Day 2 to their likely failure to reach a pharmacologically significant BAL after Test Day 1. Thus, while HDID mice are relatively insensitive to an EtOH-induced CTA, they can develop one to a self-administered dose of 2 g/kg or greater in a very short session (20 min).

The final experiment took advantage of the finding that increasing availability of EtOH sources can enhance voluntary ingestion. Offering even a second tube of 20% EtOH led to initial intakes of 4–5 g/kg, while mice offered a single tube ingested about 2.0 g/kg (See Figure 6). A clear CTA was seen in those mice drinking the greater amount on Day1 from multiple tubes. Our mice were in no way fluid restricted, and we don’t know why mice ingest more EtOH when two or three tubes are available. The phenomenon is clearly not specific to alcohol solutions but extends to other tastant solutions, as well as food. In their studies demonstrating this phenomenon in mice and rats, Tordoff and Bachmanov offer some possible reasons, among which they seem to favor the ethologically-framed suggestion that sampling from a variety of available sources may be evolutionarily advantageous [41,53,54]. In their work, they point out that when more than one fluid choice is offered, both the absolute and the relative-to-total availability must be controlled. Whatever the mechanism, the manipulation clearly works.

We found little evidence of systematic sex differences in these experiments. In three experiments, we included both sexes. While the numbers of mice we studied were sufficient to yield strong inferences about any main effects of sex, they were marginal for detecting interactive effects of sex with other variables such as genotype or treatment [55]. However, in those studies (Experiments 1, 3, and 4) we found the occasional significant interaction with sex, but saw upon further scrutiny no suggestion that it might prove biologically informative with increased numbers of subjects. In Experiment 5, we studied only males, and the published multiple bottle experiments were also limited to males [41,53,54], so we do not know whether this finding will extrapolate to females. The larger question is whether sex differences in sensitivity to develop or perform a CTA to EtOH may characterize the iHDID mouse lines. Our Experiment 1 saw little support for this possibility, but sex has certainly been shown to play a role in adult and adolescent rats in some studies [56,57]. We consider this an area needing further investigation.

## 5. Conclusions

In summary, these experiments suggest that HDID mice are willing to drink EtOH solutions to the point of intoxication in part due to their genetic insensitivity to the aversive effects of ethanol. This insensitivity has emerged as a correlated response to selection. It is maintained in both inbreds derived from the original selected lines, and appears to be more pronounced in iHDID-1 than iHDID-2. These experiments also show that offering higher concentrations of EtOH or making it more available can enhance both the intake and the BAL achieved from voluntary drinking. These animals are available to interested investigators upon request. 

## Supplementary Materials

The following are available online at http://www.mdpi.com/2076-3425/9/1/2/s1, Figure S1: Intake across days of EtOH taste aversion conditioning in females and males, Figure S2: Day 2 intakes and blood alcohol levels after different EtOH concentrations.

## References

[1] Olsson C.A., Romaniuk H., Salinger J., Staiger P.K., Bonomo Y., Hulbert C., Patton G.C. (2016). Drinking Patterns of Adolescents Who Develop Alcohol Use Disorders: Results from the Victorian Adolescent Health Cohort Study. BMJ Open.

[2] https://www.samhsa.gov/data/sites/default/files/NSDUH-FFR1-2016/NSDUH-FFR1-2016.htm.

[3] https://www.niaaa.nih.gov/alcohol-health/overview-alcohol-consumption/alcohol-facts-and-statistics.

[4] National Institute on Alcohol Abuse and Alcoholism (NIAAA) (2004). NIAAA Council Approves Definition of Binge Drinking.

[5] Rolland B., Naassila M. (2017). Binge Drinking: Current Diagnostic and Therapeutic Issues. CNS Drugs.

[6] Samson H.H., Files F.J., Denning C., Marvin S. (1998). Comparison of alcohol-preferring and nonpreferring selectively bred rat lines. I. Ethanol initiation and limited access operant self- administration. Alcohol. Clin. Exp. Res..

[7] Oberlin B., Best C., Matson L., Henderson A., Grahame N. (2011). Derivation and characterization of replicate high- and low-alcohol preferring lines of mice and a high-drinking crossed HAP line. Behav. Genet..

[8] Crabbe J.C., Noronha A.B.C., Cui C., Harris R.A., Crabbe J.C. (2014). The genetic complexity of alcohol drinking in rodents. The Neurobiology of Alcohol Dependence.

[9] Petkov P.M., Ding Y., Cassell M.A., Zhang W., Wagner G., Sargent E.E., Asquith S., Crew V., Johnson K.A., Robinson P. (2004). An efficient SNP system for mouse genome scanning and elucidating strain relationships. Genome Res..

[10] Dole V.P., Gentry R.T. (1984). Toward an analogue of alcoholism in mice: Scale factors in the model. Proc. Natl. Acad. Sci. USA.

[11] Esser M.B., Hedden S.L., Kanny D., Brewer R.D., Gfroerer J.C., Naimi T.S. (2014). Prevalence of alcohol dependence among US adult drinkers, 2009–2011. Prev. Chronic Dis..

[12] Humphreys K., Bickel W.K. (2018). Toward a neuroscience of long-term recovery from addiction. JAMA Psychiatry.

[13] Verendeev A., Riley A.L. (2013). The role of the aversive effects of drugs in self-administration: Assessing the balance of reward and aversion in drug-taking behavior. Behav. Pharmacol..

[14] Revusky S. (1973). Some laboratory paradigms for chemical aversion treatment of alcoholism. J. Behav. Ther. Exp. Psychiatry.

[15] Nathan P.E. (1985). Aversion therapy in the treatment of alcoholism: Success and failure. Ann. N. Y. Acad. Sci..

[16] Riley A.L. (2011). The paradox of drug taking: The role of the aversive effects of drugs. Physiol. Behav..

[17] Elkins R.L. (1986). Separation of taste-aversion-prone and taste-aversion-resistant rats through selective breeding: Implications for individual differences in conditionability and aversion-therapy alcoholism treatment. Behav. Neurosci..

[18] Orr T.E., Whitford-Stoddard J.L., Elkins R.L. (2004). Taste-aversion-prone (TAP) rats and taste-aversion-resistant (TAR) rats differ in ethanol self-administration, but not in ethanol clearance or general consumption. Alcohol.

[19] Orr T.E., Walters P.A., Elkins R.L. (1997). Differences in free-choice ethanol acceptance between taste aversion-prone and taste aversion-resistant rats. Alcohol. Clin. Exp. Res..

[20] Elkins R.L., Walters P.A., Orr T.E. (1992). Continued development and unconditioned stimulus characterization of selectively bred lines of taste aversion prone and resistant rats. Alcohol. Clin. Exp. Res..

[21] Green A.S., Grahame N.J. (2008). Ethanol drinking in rodents: Is free-choice drinking related to the reinforcing effects of ethanol?. Alcohol.

[22] Spierling S.R., Kreisler A.D., Williams C.A., Fang S.Y., Pucci S.N., Kines K.T., Zorrilla E.P. (2018). Intermittent, extended access to preferred food leads to escalated food reinforcement and cyclic whole-body metabolism in rats: Sex differences and individual vulnerability. Physiol. Behav..

[23] Zorrilla E.P., Inoue K., Fekete E.M., Tabarin A., Valdez G.R., Koob G.F. (2005). Measuring meals: Structure of prandial food and water intake of rats. Am. J. Physiol. Regul. Integr. Comp. Physiol..

[24] Linsenbardt D.N., Boehm S.L. (2014). Alterations in the rate of binge ethanol consumption: Implications for preclinical studies in mice. Addict. Biol..

[25] Rhodes J.S., Best K., Belknap J.K., Finn D.A., Crabbe J.C. (2005). Evaluation of a simple model of ethanol drinking to intoxication in C57BL/6J mice. Physiol. Behav..

[26] Thiele T.E., Navarro M. (2014). “Drinking in the dark” (DID) procedures: A model of binge-like ethanol drinking in non-dependent mice. Alcohol.

[27] Fritz B.M., Boehm S.L. (2016). Rodent models and mechanisms of voluntary binge-like ethanol consumption: Examples, opportunities, and strategies for preclinical research. Prog. Neuropsychopharmacol. Biol. Psychiatry.

[28] Jeanblanc J., Rolland B., Gierski F., Martinetti M.P., Naassila M. (2018). Animal models of binge drinking, current challenges to improve face validity. Neurosci. Biobehav. Rev..

[29] Crabbe J.C., Metten P., Rhodes J.S., Yu C.-H., Brown L.L., Phillips T.J., Finn D.A. (2009). A line of mice selected for high blood ethanol concentrations shows drinking in the dark to intoxication. Biol. Psychiatry.

[30] Crabbe J.C., Metten P., Belknap J.K., Spence S.E., Cameron A.J., Schlumbohm J.P., Huang L.C., Barkley-Levenson A.M., Ford M.M., Phillips T.J. (2014). Progress in a replicated selection for elevated blood ethanol concentrations in HDID mice. Genes Brain Behav..

[31] Barkley-Levenson A.M., Crabbe J.C. (2015). Distinct ethanol drinking microstructures in two replicate lines of mice selected for drinking to intoxication. Genes Brain Behav..

[32] Crabbe J.C., Gerlai R.T. (2018). Using Signatures of Directional Selection to Guide Discovery. Molecular-Genetic and Statistical Techniques for Behavioral and Neural Research.

[33] Barkley-Levenson A.M., Crabbe J.C. (2014). High drinking in the dark mice: A genetic model of drinking to intoxication. Alcohol.

[34] Cunningham C.L., Phillips T.J., Maldonado R. (2003). Genetic Basis of Ethanol Reward. Molecular Biology of Drug Addiction.

[35] Stephens D.N., Duka T., Crombag H.S., Cunningham C.L., Heilig M., Crabbe J.C. (2010). Reward sensitivity: Issues of measurement, and achieving consilience between human and animal phenotypes. Addict. Biol..

[36] Barkley-Levenson A.M., Cunningham C.L., Smitasin P.J., Crabbe J.C. (2015). Rewarding and aversive effects of ethanol in High Drinking in the Dark selectively bred mice. Addict. Biol..

[37] Broadbent J., Muccino K.J., Cunningham C.L. (2002). Ethanol-induced conditioned taste aversion in 15 inbred mouse strains. Behav. Neurosci..

[38] Barkley-Levenson A.M., Crabbe J.C. (2012). Ethanol drinking microstructure of a High Drinking in the Dark selected mouse line. Alcohol. Clin. Exp. Res..

[39] Belknap J.K., Coleman R.R., Foster K. (1978). Alcohol consumption and sensory threshold differences between C57BL/6J and DBA/2J mice. Physiol. Psychol..

[40] McClearn G.E., Arnold W.J. (1968). Genetics and motivation of the mouse. Nebraska Symposium on Motivation.

[41] Tordoff M.G., Bachmanov A.A. (2003). Influence of the number of alcohol and water bottles on murine alcohol intake. Alcohol. Clin. Exp. Res..

[42] Finn D.A., Belknap J.K., Cronise K., Yoneyama N., Murillo A., Crabbe J.C. (2005). A procedure to produce high alcohol intake in mice. Psychopharmacology.

[43] Ryabinin A.E., Sharpe A.L., Tsivokovskaia N.O., Weitemier A.Z. (2003). Ethanol self-administration during the circadian dark phase. Alcohol. Clin. Exp. Res..

[44] National Research Council (2010). Guide for the Care and Use of Laboratory Animals.

[45] Pardoe I. (2012). Applied Regression Modeling.

[46] Falconer D.S., Mackay T.F.C. (1996). Introduction to Quantitative Genetics.

[47] Lynch M., Walsh B. (1998). Genetics and Analysis of Quantitative Traits.

[48] Eisen E.J., Hanrahan J.P., Legates J.E. (1973). Effects of population size and selection intensity on correlated responses to selection for postweaning gain in mice. Genetics.

[49] Sharpe A.L., Tsivkovskaia N.O., Ryabinin A.E. (2005). Ataxia and c-Fos expression in mice drinking ethanol in a limited access session. Alcohol. Clin. Exp. Res..

[50] McClearn G.E., Rodgers D.A. (1959). Differences in alcohol preference among inbred strains of mice. Q. J. Stud. Alcohol.

[51] Thomas K. (1969). Selection and avoidance of alcohol solutions by two strains of inbred mice and derived generations. Q. J. Stud. Alcohol.

[52] Crabbe J.C., Spence S.E., Brown L.L., Metten P. (2011). Alcohol preference drinking in a mouse line selectively bred for high drinking in the dark. Alcohol.

[53] Tordoff M.G., Bachmanov A.A. (2003). Mouse taste preference tests: Why only two bottles?. Chem. Sens..

[54] Tordoff M.G. (2002). Obesity by choice: The powerful influence of nutrient availability on nutrient intake. Am. J. Physiol. Regul. Integr. Comp. Physiol..

[55] Wahlsten D. (1990). Insensitivity of the analysis of variance to hereditary-environment interaction. Behav. Brain Sci..

[56] Morales M., Schatz K.C., Anderson R.I., Spear L.P., Varlinskaya E.I. (2014). Conditioned taste aversion to ethanol in a social context: Impact of age and sex. Behav. Brain Res..

[57] Schramm-Sapyta N.L., Francis R., MacDonald A., Keistler C., O’Neill L., Kuhn C.M. (2014). Effect of sex on ethanol consumption and conditioned taste aversion in adolescent and adult rats. Psychopharmacology.

