# Supplementary material for: Ethanol Conditioned Taste Aversion in High Drinking in the Dark Mice
Source: Brain Sci. 2019 Jan 1;9(1):2. doi: 10.3390/brainsci9010002 (PMC6356868; doi:10.3390/brainsci9010002)
Supplement: Supplementary file 1 [file brainsci-09-00002-s001.zip › captions.pdf]

Supplemental Figure 1. Intake across days of EtOH taste aversion conditioning varies as a function of genotype and dose. Panel a shows means ± SEM for groups of 6-11 female mice, and Panel b for groups of 5-12 males. Compare with Figure 1 in the main text, which shows the same data for the sexes combined.

Supplemental Figure 2. Mean ± SEM intake (Panel a) and BALs (Panel b) after 4 hr on Day 2 for groups of 23-24 mice per genotype and concentration, collapsed on sex.
